# Relationship between potentially inappropriate medications and functional prognosis in elderly patients with distal radius fracture: a retrospective cohort study

**DOI:** 10.1186/s13018-020-01861-w

**Published:** 2020-08-12

**Authors:** Takako Nagai, Masahiro Nagaoka, Koji Tanimoto, Yoshiaki Tomizuka, Hiroshi Uei, Kazuyoshi Nakanishi

**Affiliations:** 1 Department of Orthopedic Surgery, Nihon University Hospital, Tokyo, Japan; 2 Department of Orthopedic Surgery, Osumi Hospital, Tokyo, Japan; 3 Department of Rehabilitation Medicine, Nihon University Hospital, 1-6 Kandasurugadai, Chiyoda-ku, Tokyo, 1018309 Japan; 4 Department of Orthopedic Surgery, Nihon University School of Medicine, Tokyo, Japan

**Keywords:** Distal radius fracture, Falls, Potentially inappropriate medications, Subsequent falls

## Abstract

**Background:**

Potentially inappropriate medications (PIMs) are a major concern in geriatric care. PIMs increase the risk of falls in elderly patients. However, the relationship between PIMs, subsequent falls, and functional prognosis for distal radius fracture (DRF) remains unclear. The aim of this study was to examine the relationship between PIMs, activities of daily living, and subsequent falls in elderly DRF patients.

**Methods:**

The study included 253 patients aged ≥ 65 years who required surgical treatment for DRF. Clinical characteristics of patients obtained included age, sex, body mass index, number of medicines used at admission, number and type of PIMs used at admission, bone mineral density, use of drugs for osteoporosis, severity of comorbidities, nutritional status, Barthel Index (BI), length of hospital stay, subsequent falls, fracture type, and Mayo wrist score. Subjects were divided into two groups according to PIMs use and no use. Propensity score matching was used to assess patient characteristics and confirm factors affecting BI and subsequent falls.

**Results:**

One hundred seven patients (42.3%) were prescribed PIMs upon hospital admission. The mean BI gain was significantly lower in patients prescribed PIMs than in those who were not (*p* = 0.006), as was the rate of falls post-surgery (*p* = 0.009). Multivariate analysis of BI gain showed that PIMs affected BI gain (95% confidence interval [CI], − 1.589 to − 0.196, *p* = 0.012), and logistic regression analysis revealed that PIMs influenced subsequent falls (odds ratio, 0.108, 95% CI, 1.246 to 2.357, *p* < 0.001).

**Conclusions:**

PIM use hindered the improvement in activities of daily living and increased the incidence of subsequent falls in patients assessed. These results demonstrate the importance of appropriate drug control for patients with DRF.

## Background

Distal radius fractures (DRF) are a common type of orthopedic fracture that results from falls in elderly individuals [1–3]. As the life span of the general population has increased, so has the incidence of DRF in the elderly [4]. While the incidence of hip and shoulder fractures increases after the age of 80, the incidence of DRFs increases at age 60–70 and therefore affects more active elderly individuals [5]. Lee et al. [6] previously reported that patients with DRF do not have significantly lower average lean body mass but that bone mineral density (BMD) is significantly lower in patients with DRF. Some reports have suggested that low body mass is a risk for hip fracture [7, 8] and that patients with DRF have a preserved body mass compared with patients with hip fracture. Optimal treatment strategies appropriate for different types of DRFs and patient categories are currently a subject of debate. Not only has the overall rate of surgically managed fractures increased, there has also been a significant increase in the use of internal plate fixation [9]. Reduced physical activity due to pain, impaired function, and fear of falling after fall-associated DRF likely increases future fall risk by decreasing the effectiveness of protective responses via the deterioration of muscular strength, balance, and reaction time [10]. In addition, elderly patients tend to have developed polypharmacy to treat multiple diseases, and it has been reported that polypharmacy is a risk factor for falls [11]. The frequency of adverse drug reactions has been shown to increase when six or more drugs are prescribed, and the frequency of falls among outpatients increases when five or more drugs are prescribed. Maki et al. investigated the effect of polypharmacy on the occurrence of hip fractures in patients who took five or more oral medications and reported that patients experienced longer hospital stays and had decreased Barthel Index (BI) efficiency, relative to those taking fewer drugs [12]. Potentially inappropriate medications (PIMs) are a major concern in geriatric care. PIMs increase the risk of falls, emergency department visits, and unplanned hospitalizations in elderly patients. Masumoto et al. [13] reported that 32.3% of elderly people with chronic diseases use PIMs, and PIMs and polypharmacy increase the risk of falls.

However, no reports have investigated the effects of polypharmacy or PIMs on the rates of DRF, despite the fact DRFs among the elderly often occur as a result of falls. Therefore, identification of unnecessary and potentially hazardous drugs among DRF patients will be useful for improving patient outcomes. Further, despite the high risk of PIM administration among these patients, the effects of treatment on activities of daily living (ADL) and adverse events have not been investigated. Therefore, the aim of the present study was to assess the association between PIMs and subsequent falls in elderly patients with DRF.

## Methods

### Study design and participants

This retrospective cohort study involved 253 patients aged 65 years or older that were admitted to an acute care hospital for surgical treatment of DRF between October 2014 and December 2018, and who were followed-up for at least 1 year post-surgery. Patients were retrospectively identified via a surgical database search that encompassed two affiliated hospitals. Demographic and postoperative clinical course information was extracted from each patient’s electronic medical record. Patients with neurological/cognitive impairment, multiple fractures, death, and missing data, including bone density information, post-spine surgery were excluded. Ethical approval was obtained from each hospital’s ethics board. Patient informed consent was waived due to the retrospective design of the study.

### Surgical treatment and rehabilitation

A volar locking plate was used for internal fixation in all cases (ACU-LOC plate, ACUMED, LLC., USA, *n* = 96 cases; Anatomic Volar Plate System, Depuy Synthes, Johnson & Johnson. Co., USA, *n* = 81 cases; Stellar2, HOYA Technosurgical Co., Japan, *n* = 47 cases; and APUTUS 2.5, Medical engineering system Co., Japan, *n* = 29 cases). Surgery was performed in all cases with a brachial plexus block. A standard volar approach was used to expose the fractured side. The fracture was approached from the radial side of the flexor carpi radialis, and the quadrate pronator muscle was incised to reduce the fracture. If the fracture was unstable, it was reduced with a Kirschner wire. Following surgery, all patients were casted for 3–7 days depending on the stability of the fracture site. Throughout postoperative rehabilitation, finger excursion training was initiated 1 day post-operation. Active and passive training of the wrist joint with one-on-one guidance began after cast removal. The rehabilitation period was defined as the 5 months that followed surgery. Evaluations were carried out by performing periodic medical examinations after the rehabilitation period had ended.

### Measurements

Data including patient age, sex, body mass index, total number of medicines used at admission, number and type of PIMs used at admission, bone mineral density (as a percentage of the mean among young adults), use of drugs for osteoporosis, fracture type, serum albumin level, comorbidity severity (Charlson Comorbidity Index [CCI]), BI, BI gain, Mayo score, length of hospital stay, and occurrence of subsequent falls were reviewed for each patient. Osteoporosis was defined as a *T*-score less than and equal to − 2.5 SD at L2–4 lumber vertebrae. Fracture type was classified using the AO/OTA classification system. This classification system is commonly used for the radiographic classification of DRF into the following three categories: A, B, and C. Category A includes extra-articular fractures, B includes intra-articular fractures, and C contains intra-articular complete fractures [14]. Comorbidity was assessed using the CCI [15]. CCI is an indicator of multi-disease comorbidities and includes diabetes with chronic complications, heart failure, kidney disease, liver disease, chronic lung disease, dementia, hemiplegia or paraplegia, malignancy, and AIDS/HIV. The CCI uses a weighted score for each comorbidity, with higher numbers indicating a greater number of comorbidities and greater risk of mortality. ADL were evaluated using BI scores. BI scores are determined through an assessment of 10 features: eating, moving, dressing, toilet movement, bathing, walking, going up and down stairs, changing clothes, defecation, and urination. Each item is scored as follows: 0, unable to complete; 1, needs help; or 2, independent. Total scores are multiplied by 20, to produce a maximum score of 100. ADL scores were assessed both before surgery and at the final follow-up appointment. BI gains were determined by subtracting preoperative BI from BI scores at the end of follow-up. The Mayo wrist score was used to evaluate wrist function. This score ranges from 0 to 100 points, and greater scores indicate increased function. The score incorporates pain scores, functional status, range of motion, and grip strength. Two criteria for PIMs are used globally: Beers criteria [16] and the Screening Tool of Older Persons’ Prescriptions (STOPP) [17]. In Japan, the Screening Tool for Older Persons’ Appropriate Prescriptions for Japanese (STOPP-J) is used [18]. Therefore, we used STOPP-J to classify PIMs in this study. PIMs were used if patients were taking STOPP-J drugs at the time of admission and continued to take the drugs 1 year after surgery.

Regarding the measurement of outcomes, the primary outcome considered was the occurrence of subsequent fall(s), and the secondary outcome considered was BI gain.

### Statistical analysis

For statistical analysis, the patients were divided into two groups: those who used PIMs and those who did not. The unpaired *t* test, Mann-Whitney’s *U* test, and *χ*2 test were used to perform comparisons between groups depending on variables assessed and the normality of data. In addition, propensity score matching was carried out. Propensity scores were calculated using age, sex, CCI, fracture type, and Mayo wrist score. Variance inflation factor (VIF) was calculated as an index of multicollinearity, and items with VIF values of 2 or less were used as independent variables. Spearman’s rank correlation was used for the univariate analysis. A multiple linear regression analysis after propensity score matching was performed to assess BI gain. Data were analysed using SPSS version 25 (IBM Corporation; Armonk, NY, USA).

### Results

**Fig. 1:**
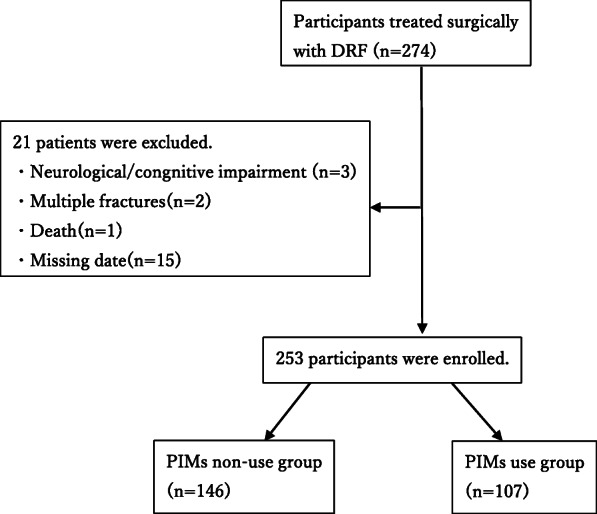
Flow-chart of patients selection

The study included 253 patients aged 65 years or older that were admitted to an acute care hospital for surgical treatment of DRF between October 2014 and December 2018, who were followed-up for at least 1-year post-surgery. Three cases involving neurological/cognitive impairment, two cases involving multiple fractures, 1 case in which the patient had died, and 15 cases that lacked necessary data were excluded from the study (Fig. 1). The subjects were divided into two groups: a PIM use group and a PIM non-use group. One hundred and seven cases (42.3%) were prescribed PIMs. Significant differences (*p* < 0.05) between the two groups were observed regarding fracture type, serum albumin level, CCI, length of hospital stay, and fall rate. Propensity score (PS) was calculated from age, sex, fracture type, CCI, and Mayo score. After PS matching, 107 cases within the PIM use group and 146 from the PIM non-use group were assessed (Table 1). Univariate analysis after PS matching revealed that the two groups significantly differed with regard to CCI, number of drugs used upon admission, BI gain, and subsequent fall rate. No significant difference was observed between the two groups with regard to the occurrence of surgical complications after DRF (Table 2). The types and distribution of PIMs are shown in Table 3. The three most frequently prescribed STOPP-J PIMs were hypotonics (24.2%), NSAIDS (16.8%), and diuretics (12.1%). Spearman’s rank correlation results are shown in Table 4. PIM use positively correlated with CCI, total number of drugs used at admission, and fall rate during follow-up periods and negatively correlated with BI gain. Results of multivariate analysis of BI gain after propensity score matching are shown in Table 5. The number of PIMs significantly affected the BI gain (PIMs, *β* = − 0.181; 95% CI, − 1.589 to − 0.196; *p* = 0.012). The results of the logistic regression analysis are shown in Table 6. The incidence of subsequent falls was correlated with the number of PIMs (odds ratio, 0.108; 95% CI, 1.246 to 2.357; *p* < 0.001).

**Table 1:** Demographic and clinical characteristics before and after matching

Characteristics	All patients (*n* = 253)			Propensity-matched patients (*n* = 191)		
	PIMs use group (*n* = 107)	PIM non-use group (*n* = 146)	*p*	PIMs use group (*n* = 107)	PIMs non-use group (*n* = 84)	*p*
Age (year)	75.6 ± 8.6	72.8 ± 7.7	0.114^1)^	75.6 ± 8.6	73.4 ± 7.7	0.112^1)^
Sex, female	93 (86.6)	124 (85.3)	0.718^2)^	93 (86.6)	72 (81.6)	0.395^2)^
Fracture type, *n* (%)			0.019^2)^			0.110^2)^
AO type A	10 (9.3)	3 (2.1)		10 (9.3)	2 (2.2)	
Type B	42 (39.3)	52 (35.6)		42 (39.3)	40 (46.0)	
Type C	55 (51.4)	91 (62.3)		55 (51.4)	42 (48.3)	
BMI (kg/m^2^)	22.4 ± 3.7	22.2 ± 3.8	0.842^1)^	22.4 ± 3.7	22.2 ± 3.6	0.561^1)^
Serum albumin (g/dl)	4.03 ± 0.44	4.09 ± 0.32	< 0.001^1)^	4.03 ± 0.44	4.07 ± 0.32	0.326^1)^
BMD (g/cm^2^)	0.89 ± 0.15	0.89 ± 0.15	0.921^1)^	0.89 ± 0.15	0.89 ± 0.14	0.476^1)^
CCI	0.57 ± 0.70	0.57 ± 0.70	< 0.001^1)^	0.57 ± 0.70	0.30 ± 0.57	0.01^1)^
Total number of drugs administered on admission	5.45 ± 2.72	5.45 ± 2.72	0.178^1)^	5.45 ± 2.72	3.41 ± 2.62	< 0.001^1)^
Use of drugs for osteoporosis, *n* (%)	20 (18.7)	22 (15.1)	0.444^2)^	20 (18.7)	12 (14.3)	0.418^2)^
Length of hospital stay	2.29 ± 2.06	1.76 ± 0.89	0.006^1)^	2.29 ± 2.06	1.83 ± 0.91	0.061^1)^
BI score						
Admission	77.2 ± 7.89	77.8 ± 6.08	0.059^1)^	77.2 ± 7.89	77.3 ± 6.41	0.720^1)^
1 year after surgery	85.3 ± 9.03	87.4 ± 8.29	0.747^1)^	85.3 ± 9.03	87.3 ± 8.75	0.117^1)^
BI gain	9.66 ± 5.80	8.04 ± 4.94	0.183^1)^	9.66 ± 5.80	10.0 ± 5.60	0.006^1)^
Mayo wrist score						
1 year after surgery	83.1 ± 6.72	84.7 ± 6.40	0.060^1)^	83.1 ± 6.72	84.7 ± 6.40	0.132^1)^
Subsequent falls, *n* (%)	24 (22.4)	13 (8.9)	0.003^2)^	24 (22.4)	7 (8.0)	0.009^2)^

Values are presented as mean ± standard, deviation or number (%), or median (interquartile range)

*PIMs* potentially inappropriate medications, *BMI* body mass index, *BMD* body mineral density, *CCI* Charlson Comorbidity Index, *BI* Barthel Index

^1)^Student *t* test

^2)^Chi-squared test

**Table 2:** Postoperative complications of DRF after matching

Complications	All (***n*** = 191)	PIMs non-use (***n*** = 84)	PIMs use (***n*** = 107)	***p*** value
				0.559^1)^
EPL rupture	1 (0.5)	0 (1.3)	1 (0.9)	
Screw loosening	1 (0.5)	0 (0)	1 (0.9)	
Compression	7 (3.6)	2 (2.2)	5 (4.7)	
Neuropathy	3 (1.5)	2 (2.2)	1 (0.9)	

Values are presented as number (%)

*PIMs* potentially inappropriate medications, *EPL* extensor pollicis longus

^1)^Chi-squared test

**Table 3:** Types and frequency of potentially inappropriate medications as pharmacotherapy

PIMs (drug class or generic names)	Patients (%)
Hypnotics	26 (24.2)
NSAIDs	18 (16.8)
Diuretics	13 (12.1)
Antipsychotics	8 (7.4)
Oral antidiabetic drugs	8 (7.4)
H_2_ receptor antagonist	7 (6.5)
Antithrombotic drugs	5 (4.7)
Laxative (magnesium oxide)	3 (2.8)
Overactive bladder medications	3 (2.8)
Steroids	3 (2.8)
H_1_ receptor antagonist (first generation)	2 (1.9)
Alpha-blockers	2 (1.9)
Antidepressants	2 (1.9)
Sulpiride	1 (0.9)
Beta-blockers	1 (0.9)
Antiparkinson drug	1 (0.9)
Digitalis	0
Insulin	0
Antiemetic drugs	0

**Table 4:** Spearman’s rank coefficients among different factors after matching

	Age (year)	BMI (kg/m^2^)	Serum albumin (g/dl)	PIMs	Total number of drugs administered on admission	CCI	BI at admission	BI at 1 year after surgery	BI gain	Fall during follow-up periods
Age (year)	1	0.053	− 0.213^**^	0.156^**^	0.292^**^	0.060	− 0.147^*^	− 0.128	− 0.051	0.165^*^
BMI (kg/m2)		1	0.213^**^	0.033	0.099	0.009	0.140	0.155^*^	0.077	− 0.070
Serum albumin (g/dl)			1	− 0.052	− 0.227^**^	− 0.209^**^	0.362^**^	0.598^**^	0.472^**^	− 0.393^**^
PIMs				1	0.469^**^	0.255^**^	− 0.050	− 0.140	− 0.168^*^	0.223^**^
Total number of drugs administered on admission					1	0.423^**^	− 0.146^*^	− 0.210^**^	− 0.165^*^	0.185^**^
CCI						1	− 0.134	− 0.275^**^	− 0.277^**^	0.269^**^
BI at admission							1	0.677^**^	− 0.064	− 0.157^*^
BI at 1 year after surgery								1	0.650^**^	− 0.223^**^
BI gain									1	− 0.208^**^
Fall during follow-up periods										1

*BMI* body mass index, *PIMs* potentially inappropriate medications, *CCI* Charlson Comorbidity Index, *BI* Barthel Index

*p < 0.05

**p < 0.01

**Table 5:** Liner regression analysis for BI gain after matching

Variables	***β***	95% confidence interval		***p*** value
		Lower	Upper	
PS	9.680	8.709	10.650	< 0.001
PIMs	− 0.181	− 1.589	− 0.196	0.012

PS (log-transformed propensity score) was calculated from log transformation of the propensity score for age, sex, Charlson Comorbidity Index, fracture type, and Mayo wrist score

*PIMs* potentially inappropriate medications

**Table 6:** Logistic regression analysis for subsequent fall after matching

Variables	Odds ratio	95% confidence interval		***p*** value
		Lower	Upper	
PS	1.713			0.001
PIMs	0.108	1.246	2.357	< 0.001

PS (log-transformed propensity score) was calculated from log transformation of the propensity score for age, sex, Charlson Comorbidity Index, fracture type, and Mayo wrist score

*PIMs* potentially inappropriate medications

## Discussion

This retrospective cohort study revealed two major findings regarding PIM use among elderly patients that experienced DRF. First, the prevalence of PIM prescriptions was determined to be 42.3%. Second, the number of PIMs prescribed may increase risk of subsequent falls post-DRF. To the best of our knowledge, this is the first study to determine characteristics of PIM use and assess the occurrence of subsequent falls in elderly patients with DRF.

Chukwulebe et al. [19] and Akkawi et al. [20] reported PIM use rates of 28.7% and 28.5%, respectively, among elderly individuals. On the other hand, Huang et al. [21] reported that 67.3% of patients receiving home care use PIMs. To define PIMs, some past reports, including one authored by Chukwulebe et al., used Beers criteria to define PIMs, while Akkawi et al. used STOPP/START, and Huang et al. used STOPP-J criteria. PIM use in the study by Huang et al. was determined to be higher than in other studies because the authors investigated elderly patients with comorbid conditions that had received home-based medical services. Additionally, PIMs categorized via STOPP-J criteria are associated with hospitalization and mortality in Japanese patients receiving home-based medical services. Our results indicate that the rate of PIM use is greater than rates determined by Chukwulebe et al. and Akkawi et al. The increased PIM use determined by this study is likely due to the inclusion of patients admitted to acute care hospitals with multiple specialty departments, and with other comorbidities. As a result, patients often received prescriptions from multiple departments, so the usage rate of PIMs was likely elevated. We used STOPP-J in this study to classify PIMs. STOPP-J is a clinical practice guideline and consensus statement for standard care of Japanese elderly patients [18]. A systematic review based on clinical questions and keywords determined these recommendations via the GRADE (grading of recommendations assessment, development, and evaluation) approach [21]. Beers criteria include drugs not used in Japan [16], and the STOPP criteria predict hospitalizations associated with inappropriate prescriptions [17]. STOPP-J is a scientifically based drug list that uses a systematic review and GRADE system to determine recommendations and evaluates the safety of drugs that may be less common in elderly individuals [22]. We used STOPP-J criteria because this study was conducted in elderly patients. STOPP-J criteria consist of a list of drugs that require particularly careful administration and a list of drugs that should be considered for treatment.

In this study, we were able to show that the number of PIMs prescribed was associated with the increased occurrence of subsequent falls. Regarding the relationship between PIMs and falls, Early et al. [23] reported that falls among geriatric patients were associated with the use of one or more PIMs. Nakamichi et al. [24] determined that antipsychotic drug use was positively associated with the risk of falls, and 6.1% of patients who experienced a hip fracture used antipsychotics. In the present study, patients used hypnotics (*n* = 26), antipsychotics (*n* = 8), and antidepressants (*n* = 2). Further, 3.1% of all patients and 7.4% of those using PIMs used antipsychotics. These rates were higher than those reported by Nakamichi et al. [24]; however, this could possibly be explained by the nature of our acute care general hospital, which treats a large number of patients with multiple diseases that are at increased risk of experiencing anxiety and insomnia. The results also suggest that the use of PIMs may have a greater effect on subsequent falls in DRF patients than in hip fracture patients.

Of the 34 patients with subsequent falls, the PIMs use group reported one vertebral fracture, one hip fracture, and 22 bruises, and the PIMs non-use group reported one clavicle fracture, one acute subdural hematoma, and 11 bruises. There were no cases of re-fracture or transposition at the site operated on for DRF.

Regarding the effect of PIMs on BI gain, there was initially no significant difference in BI gain of DRF between the two groups, but there was a significant difference in BI gain between the two groups after propensity score matching. Moreover, multiple linear regression analysis showed that PIMs influenced BI gain, and a negative correlation was observed between BI gain and PIM use. These findings indicate that ADL acquisition tends to decrease with increased PIM use. The number of PIMs showed a positive correlation with CCI, suggesting that increased PIM use may interfere with ADL acquisition in patients experiencing multiple morbidities. In this study, age, serum album, PIMs, BI, and CCI were identified as factors associated with subsequent falls. In addition, logistic regression analysis using propensity score matching to assess the probability of experiencing subsequent falls revealed that PIM use enhanced the probability of experiencing subsequent falls. Early et al. [23] examined the risk factors of falls in elderly individuals and found that age and number of PIMs increased the fall risk. They indicated that antipsychotics, antidepressants, psychotic medicines, opioids, and neuropathic medicines were drugs that enhanced the risk of falling. The study reported that the numbers of patients who repeatedly experienced falls after taking hypnotics, NSAIDS, and both hypnotics and NSAIDS were 10, 8, and 6, respectively. Although the types of PIMs differed from those reported by Early et al., the results of the present study suggest that the number of PIMs, in addition to the type of PIMs prescribed, may influence the occurrence of falls. After propensity score matching, there was a significant difference in the total number of medications used in the comparison of the two groups, and it was considered necessary to reduce the number of drugs in order to reduce PIMs. The results of this study show how increasing the awareness of health care staff in terms of risk factors for PIMs, and improving patient education, may increase ADL and prevent subsequent falls in patients with DRF.

This study had a few limitations. First, detailed patient data, including sarcopenia, muscle strength, and pain assessment were lacking due to the retrospective study design. Second, motor function after DRF and its association with PIMs were not assessed using the timed up and go test, or balance test. The Mayo wrist score was not significantly different from PIMs use in this study. However, there was a significant difference between the two groups in terms of fracture type, and it is necessary to examine the relationship between wrist joint function, fracture type, PIMs, and other influencing factors by increasing the number of databases analysed, thereby improving the power of the study.

## Conclusion

This study revealed that the number of PIMs prescribed affected ADL after DRF and increased the risk of experiencing subsequent falls. Therefore, appropriate PIM management at admission and the incorporation of fall prevention programs are important for improving outcomes in elderly DRF patients.

## Data Availability

The datasets used/or analyzed during the current study are available from the corresponding author on reasonable request.

## Abbreviations

BI: Barthel Index
BMI: Body mass index
BMD: Bone mineral density
CCI: Charlson Comorbidity Index
DRF: Distal radius fracture
EPL: Extensor pollicis longus
PIMs: Potentially inappropriate medications
PS: Log-transformed propensity score

## References

[1] Nellans KW, Kowalski E, Chung KC (2012). The epidemiology of distal radius fractures. Hand Clin.

[2] Franceschi F, Franceschetti E, Paciotti M, Cancilleri F, Maffulli N, Denaro V (2015). Volar locking plates versus K-wire/pin fixation for the treatment of distal radial fractures: a systematic review and quantitative synthesis. Br Med Bull.

[3] Smeraglia F, Del Buono A, Maffulli N (2016). Wrist arthroscopy in the management of articular distal radius fractures. Br Med Bull.

[4] Melton LJ, Chrischilles EA, Cooper C, Lane AW, Riggs BL (1992). How many women have osteoporosis?. J Bone Miner Res.

[5] Tang JB (2014). Distal radius fracture: diagnosis, treatment, and controversies. Clin Plast Surg.

[6] Lee JK, Yoon BH, Oh CH, Kim JG, Han SH (2018). Is sarcopenia a potential risk factor for distal radius fracture? Analysis using propensity score matching. J Bone Metab.

[7] Kim SH, Yi SW, Yi JJ, Kim YM, Won YJ (2018). Association between body mass index and the risk of hip fracture by sex and age: a prospective cohort study. J Bone Miner Res.

[8] Axelsson KF, Wallander M, Johansson H, Lundh D, Lorentzon M (2017). Hip fracture risk and safety with alendronate treatment in the oldest-old. J Intern Med.

[9] Mellstrand-Navarro C, Pettersson HJ, Tornqvist H, Ponzer S (2014). The operative treatment of fractures of the distal radius is increasing: results from a nationwide Swedish study. Bone Jt J.

[10] Cho YJ, Gong HS, Song CH, Lee YH, Baek GH (2014). Evaluation of physical performance level as a fall risk factor in women with a distal radial fracture. J Bone Joint Surg Am.

[11] Kojima T, Akishita M, Kameyama Y, Yamaguchi K, Yamamoto H, Eto M, Ouchi Y (2012). High risk of adverse drug reactions in elderly patients taking six or more drugs: analysis of inpatient database. Geriatr Gerontol Int.

[12] Maki H, Wakabayashi H, Nakamichi M, Momosaki R (2019). Impact of number of drug types on clinical outcome in patients with acute hip fracture. J Nutr Health Aging.

[13] Masumoto S, Sato M, Maeno T, Ichinohe Y, Maeno T (2018). Potentially inappropriate medications with polypharmacy increase the risk of falls in older Japanese patients: 1-year prospective cohort study. Geriatr Gerontol Int.

[14] Mulders MAM, Rikli D, Goslings JC, Schep NWL (2017). Classification and treatment of distal radius fractures: a survey among orthopaedic trauma surgeons and residents. Eur J Trauma Emerg Surg.

[15] D’Hoore W, Sicotte C, Tilquin C (1993). Risk adjustment in outcome assessmen: the Charlson comorbidity index. Methods Inf Med.

[16] Campanelli CM (2012). American Geriatrics Society updated Beers Criteria for potentially inappropriate medication use in older adults. J Am Geriatr Soc.

[17] Gallagher P, Ryan C, Byrne S, Kennedy J, O’Mahony D (2008). STOPP (Screening Tool of Older Person's Prescriptions) and START (Screening Tool to Alert doctors to Right Treatment). Consensus validation. Int J Clin Pharmacol Ther.

[18] Kojima T, Mizukami K, Tomita N, Arai H, Ohrui T, Eto M, Takeya Y, Isaka Y, Rakugi H, Sudo N, Arai H, Aoki H, Horie S, Ishii S, Iwasaki K, Takayama S, Suzuki Y, Matsui T, Mizokami F, Furuta K, Toba K, Akishita M, Working Group on Guidelines for Medical Treatment and its Safety in the Elderly (2016). Screening tool for older persons’ appropriate prescriptions for Japanese: report of the Japan Geriatrics Society Working Group on “Guidelines for medical treatment and its safety in the elderly”. Geriatr Gerontol Int.

[19] Chukwulebe SB, Kim HS, McCarthy DM, Courtney DM, Lank PM, Gravenor SJ, Dresden SM (2019). Potentially inappropriate medication prescriptions for older adults with painful conditions and association with return emergency department visits. J Am Geriatr Soc.

[20] Akkawi ME, Nik Mohamed MH, Md Aris MA (2019). Does inappropriate prescribing affect elderly patients' quality of life? A study from a Malaysian tertiary hospital. Qual Life Res.

[21] Guyatt GH, Oxman AD, Vist GE, Kunz R, Falck-Ytter Y, Alonso-Coello P, Schünemann HJ, GRADE Working Group (2008). GRADE: an emerging consensus on rating quality of evidence and strength of recommendations. BMJ..

[22] Huang CH, Umegaki H, Watanabe Y, Kamitani H, Asai A, Kanda S, Nomura H, Kuzuya M (2019). Potentially inappropriate medications according to STOPP-J criteria and risks of hospitalization and mortality in elderly patients receiving home-based medical services. PLoS One.

[23] Early NK, Fairman KA, Hagarty JM, Sclar DA (2019). Joint effects of advancing age and number of potentially inappropriate medication classes on risk of falls in medicare Enrollees. BMC Geriatr.

[24] Nakamichi M, Wakabayashi H, Nishioka S, Momosaki R (2019). Influence of antipsychotics on functional prognosis after geriatric hip fracture. J Nutr Health Aging.

